# Supplementary material for: Endocrine aryl hydrocarbon receptor signaling is induced by moderate cutaneous exposure to ultraviolet light
Source: Sci Rep. 2019 Jun 11;9:8486. doi: 10.1038/s41598-019-44862-4 (PMC6560103; doi:10.1038/s41598-019-44862-4)
Supplement: Supplementary file 1 — supplementary figures and table [file 41598_2019_44862_MOESM1_ESM.pdf]

Supplementary Information

Endocrine aryl hydrocarbon receptor signaling is induced by moderate cutaneous exposure to ultraviolet light

Babak Memari, Loan Nguyen-Yamamoto, Reyhaneh Salehi-Tabar, Michela Zago, Jorg Fritz, Carolyn J. Baglole, David Goltzman, and John H. White

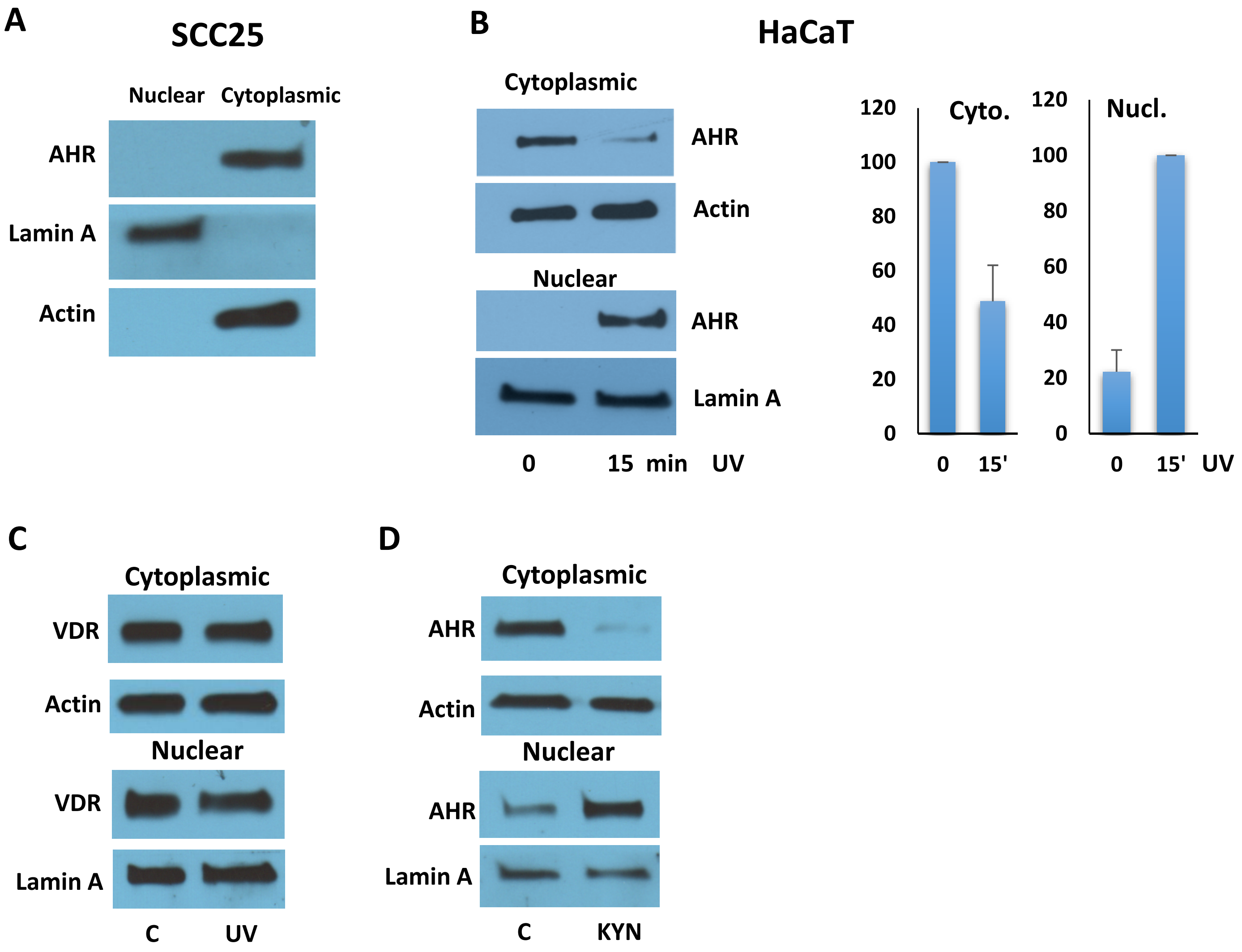

Figure S1. A) Western Blot analysis of distribution of nuclear and cytoplasmic proteins. Actin was probed as a cytoplasmic marker; lamin A was probed as a nuclear marker. AHR and the internal controls were taken from the same blot. Blot images are provided in the Supplementary Fig S6A. **B)** An analysis of the effect of a single 15 min exposure to UVB on subcellular localization of the AHR in HaCaT keratinocytes. AHR and the internal controls were taken from the same blot. Blot images are provided in the Supplementary Fig S6B. **C)** Western blot analyses of VDR protein in nuclear and cytoplasmic fractions in SCC25 cells 1 hr following irradiation with UVB (15 min). VDR and the internal controls were taken from the same blot. Blot images are provided in the Supplementary Fig S6C. **D)** Western Blot analyses of AHR from nuclear and cytoplasmic fractions in SCC25 cells, 1 hr following treatment with 50 uM Kynurenin. AHR and the internal controls were taken from the same blot. Blot images are provided in the Supplementary Fig S6D.

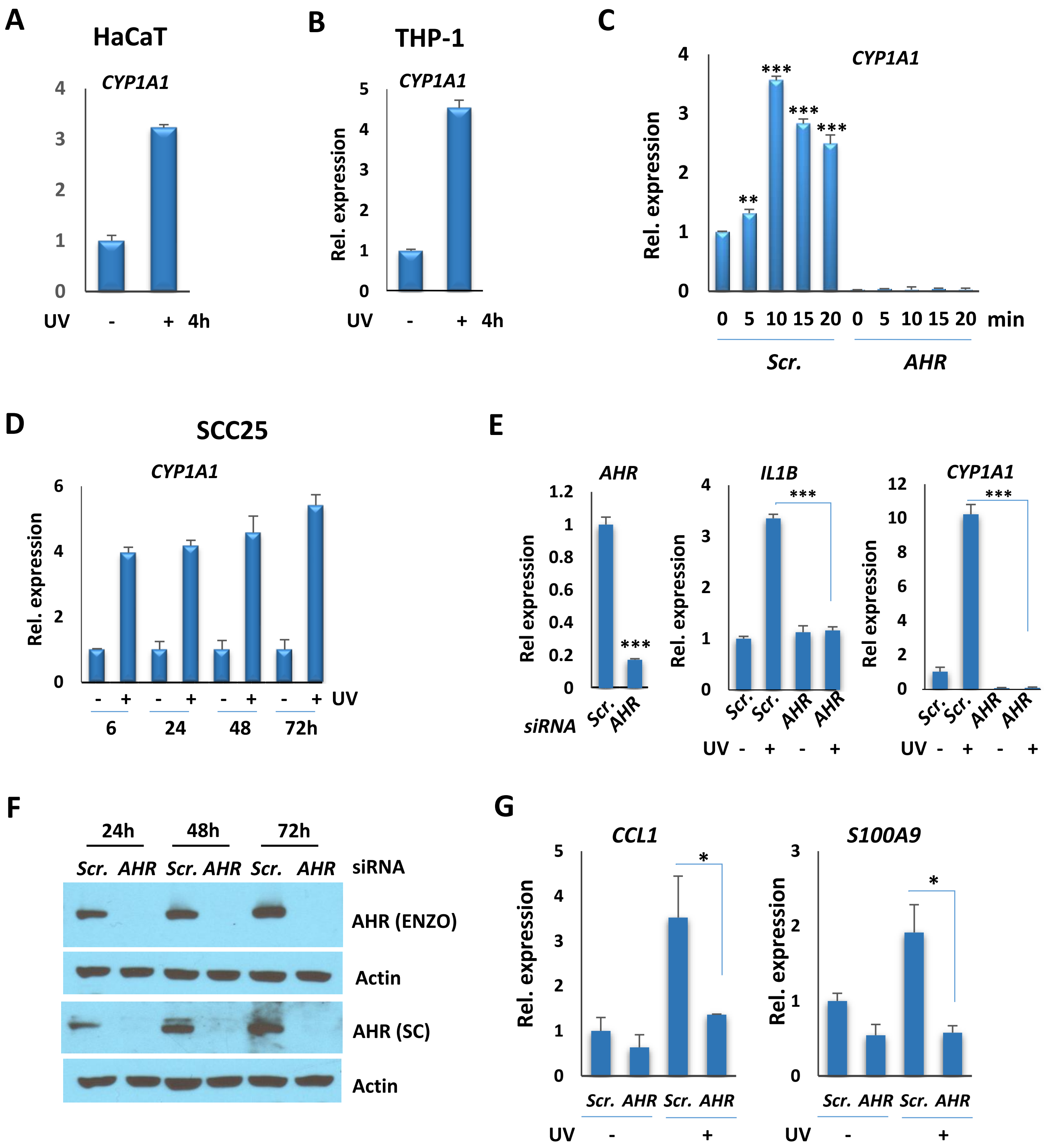

Figure S2: A, B) RT-qPCR analysis of *CYP1A1* transcription in HaCaT keratinocytes (A) and THP-1 cells (B) 4 hr following UVB exposure (15 min). **C)** . Expression of *CYP1A1* in SCC25 cells exposed to 1.2kJ/m2 of broadband UV for 0-20min, as indicated. RNA was harvested 4h after UV exposure. **D)** RT-qPCR analysis of CYP1A1 transcription in SCC25 cells 6, 24, 48, and 72 hr following irradiation with UVB (15 min). The expression of CYP1A1 in UV-exposed cells is a fold expression relative to the control cells, and is set as 1 in each time point. **E)** (left panel) RT-qPCR assay of *AHR* mRNA expression after knockdown of its gene with pooled siRNA #2 in SCC25 cells. (center and right panel) RT-qPCR analysis of *CYP1A1* and *IL1B* transcription in SCC25 cells following knockdown of the *AHR* gene and 4 hr after exposing cells to UVB for 15 min. **F)** Validation of the efficacy of pooled siRNAs against the AHR tested by western blotting after 24, 48 or 72h of knockdown with anti-AHR antibodies ENZO (BML-SA210-0100) and Santa cruz (sc-5579). AHR and Actin were taken from the same blot. Blot images are provided in the Supplementary Fig S6E. **G)** RT-qPCR analysis of *CCL1* and *S100A9* transcription in SCC25 cells following knockdown of AHR expression with siRNA #2 and 4 hr after exposing to UVB for 15 min. *P ≤ 0.05, **P ≤ 0.01, ***P ≤ 0.001 as determined by one-way ANOVAs followed by Tukey's post hoc test for multiple comparisons.

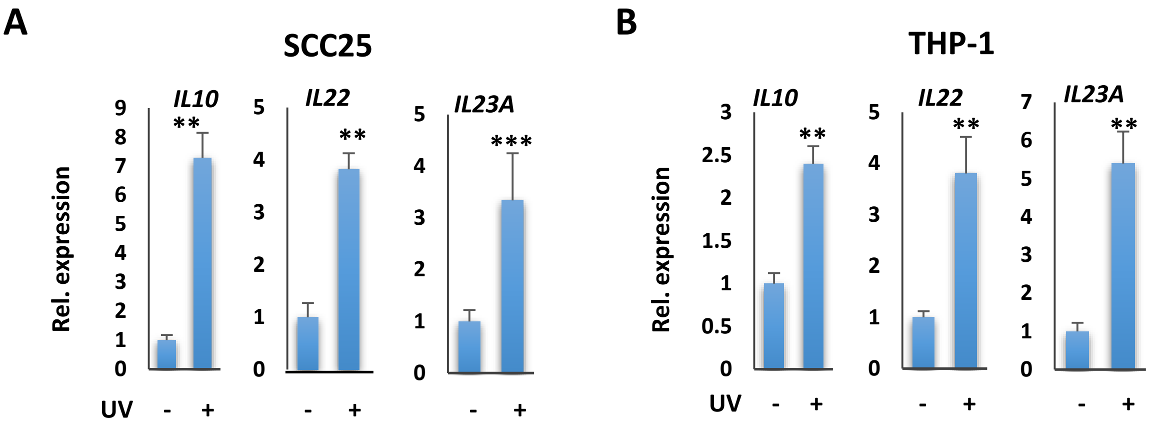

Figure S3. A) RT-qPCR assay of *IL10*, *IL22* and *IL23A* mRNAs in SCC25 cells, 4 hr after 15 min of UVB irradiation. **B)** RT-qPCR assay of *IL10*, *IL22* and *IL23A* mRNAs in THP-1 cells, 4 hr after 15 min of UVB irradiation. *P ≤ 0.05, **P ≤ 0.01, ***P ≤ 0.001 as determined by one-way ANOVAs followed by Tukey's post hoc test for multiple comparisons.

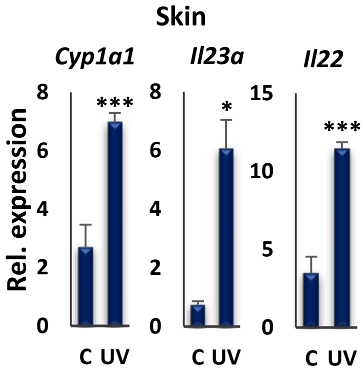

Figure S4. Effect of a single 15 min dose of cutaneous UVB irradiation on expression of AHR target genes *Cyp1a1, Il23a*, and *Il22* in mouse skin (n = 3 per group). RNA was extracted 4hr after UVB exposure.*P ≤ 0.05, ***P ≤ 0.001 as determined by one-way ANOVAs followed by Tukey's post hoc test for multiple comparisons.

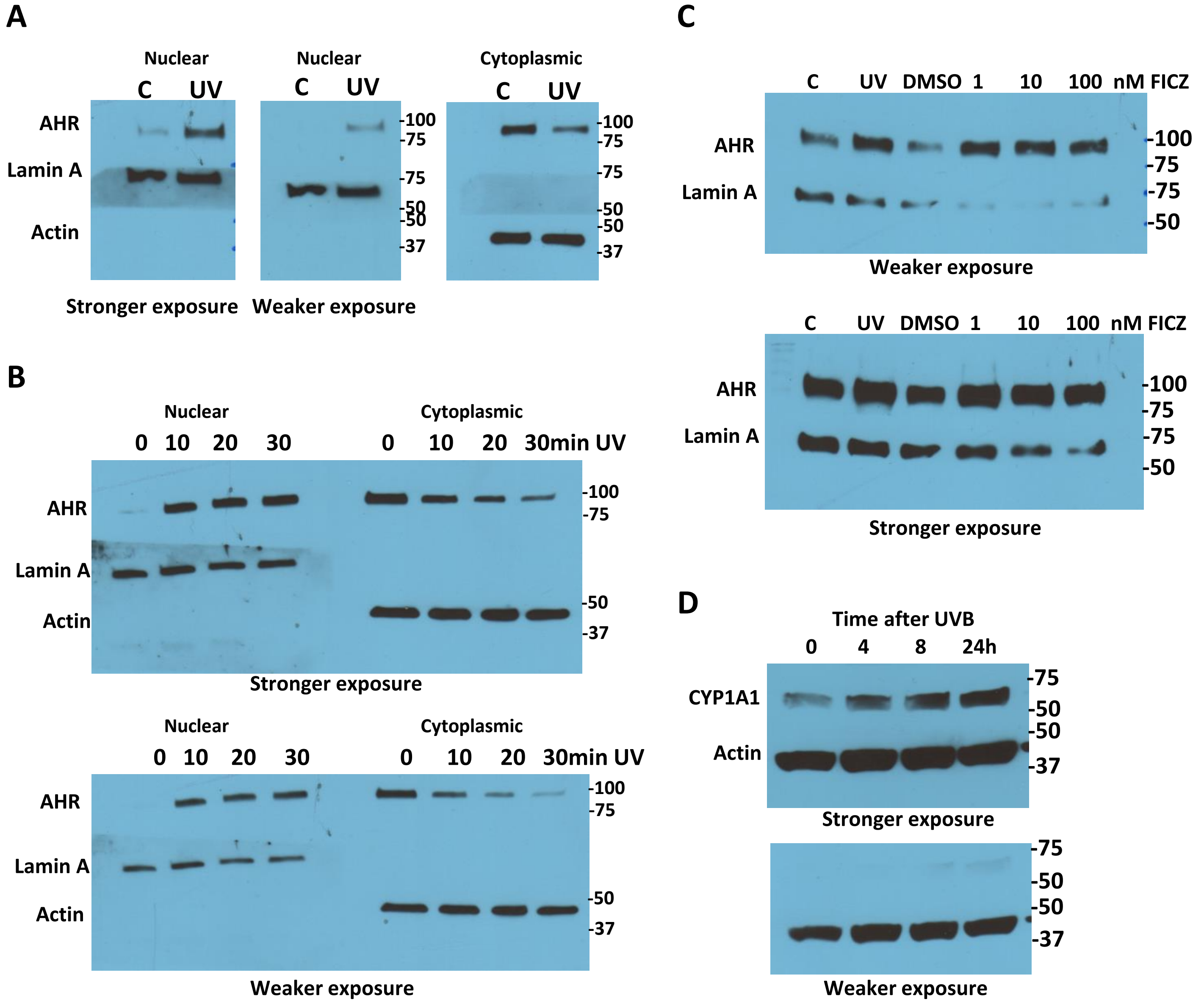

Figure S5: A) The blot corresponding to figure 1A. The blot was cut in three, up the 75 and 50 KD ladder bands. **B)** The blot corresponding to figure 1B. The blot was cut in two, up the 75 KD ladder band in the nuclear fraction part; and was cut in two, up the 50 KD ladder band in cytoplasmic part. **C)** The blot corresponding to figure 1C. The blot was cut in two, up the 75 KD ladder band. **D)** The blot corresponding to figure 1E. The blot was cut in two, at the 50 KD ladder band.

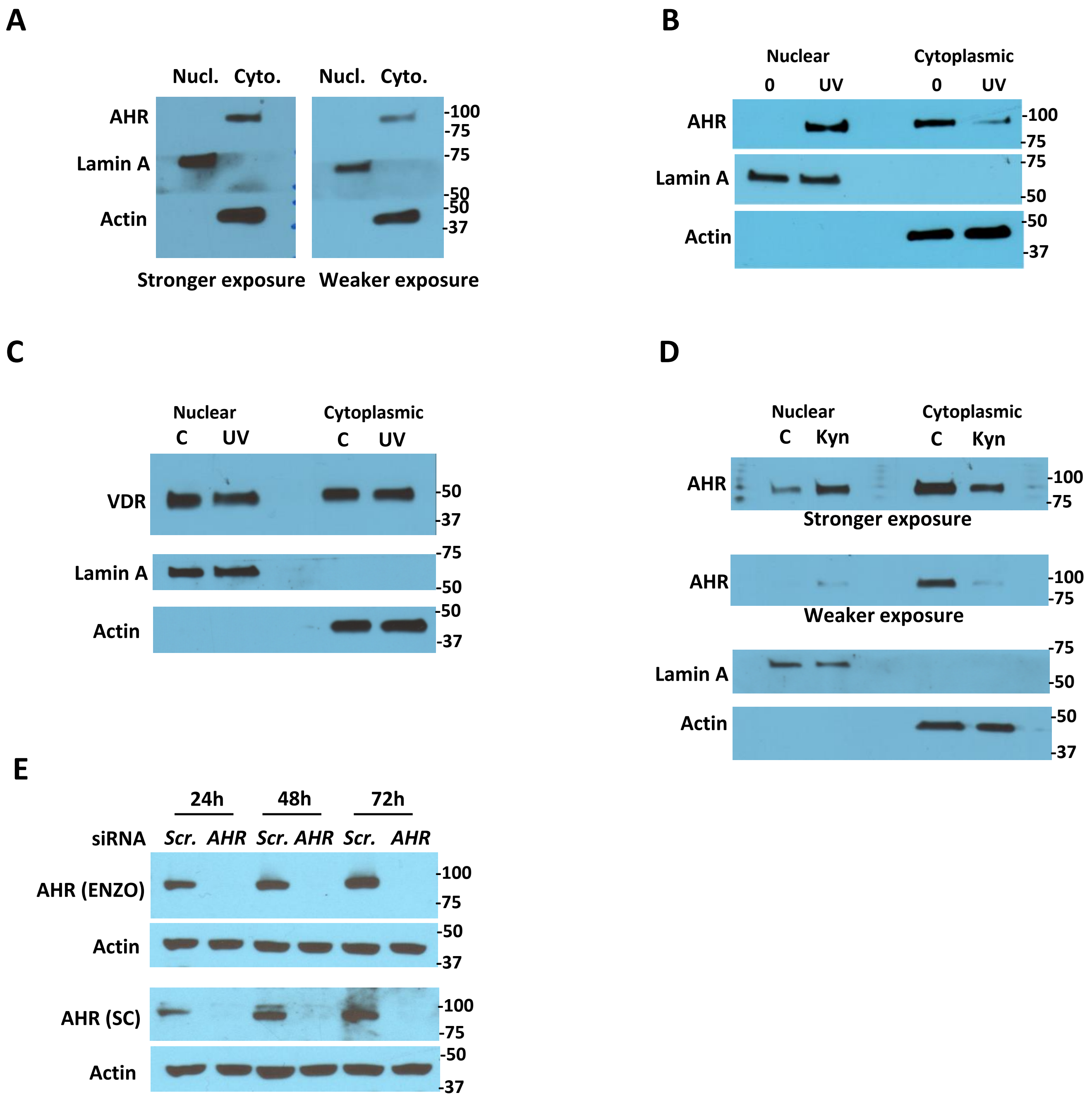

Figure S6: A) The blot corresponding to figure S1A. The blot was cut in three, up the 75 and 50 KD ladder bands. **B)** The blot corresponding to figure S1B. The blot was cut in three, up the 75 and 50 KD ladder bands. **C)** The blot corresponding to figure S1C. The blot was first probed for VDR and then stripped and cut in two, up the 75 and 50 KD ladder bands and probed for Lamin A and Actin. **D)** The blot corresponding to figure S1D. The blot was cut in three, up the 75 and 50 KD ladder bands. . **E)** The blots corresponding to figure S2F. The blots were cut in two, up the 50 KD.

Table S1: Primer sequences used for gene expression analysis		
**Human AHR**	F: CTTCCAAGCGGCATAGAGAC	R: AGTTATCCTGGCCTCCGTT
**Human IL1B**	F: AGATGAAGTGCTCCTTCCAGG	R: GGTCGGAGATTCGTAGCTGG
**Human CYP1A1**	F: CTTGGAACCTTCCCTGATCCTTG	R: GATCTTGGAGGTGGCTGAGGTA
**Human actin (ATCB)**	F: CACCTTCACCGTTCCAGTTTT	R: AACCTAACTTGCGCAGAAAACAA
**Human RNA18S5**	F: ACCCGTTGAACCCCATTCGTGA	R: GCCTCACTAAACCATCCAATCGG
**Mouse Hmbs**	F: GTGCCTACCATACTACCTCCTG	R: ACTCTCCTCAGAGAGCTGGTTC
**Mouse Ahr**	F: TGATGCCAAAGGGCAGCTTA	R: TGAACTGGTACCCCGATCCT
**Mouse Il23a**	F: ACCAGCGGGACATATGAATCT	R: AGACCTTGGCGGATCCTTTG
**Mouse Il22**	F: CTGTTGACACTTGTGCGATCTCTG	R: TTGACGGGCAGCGCATTTG
**Mouse Nqo1**	F: GATGGGAGGTACTCGAATCTGAC	R: AGCTCACCTGTGATGTCATTTCT
**Mouse Cyp1a1**	F: TTTAAAACACGCCCGCTGTG	R: CAGGCACAATGTCCCAGGAT
**Primer sequences used for ChIP analysis**		
**Human AHR on IL23A**	F:GTCAGTTGTAGCCCTGGATGTA	R:TTGGGTAGGAAGAAGGGTTGGT
**Human AHR on CYP1A1 (ref 1)**	F: GCGCGAACCTCAGCTAGT	R:TTCCCGGGGTTACTGAGTC
**Human AHR on IL10 (ref 2)**	F: GTCTTGGGTATTCATCCCAGGTTGGGG	R:CTGTGGGTTCTCATTCGCGTGTTCCTA
**Mouse AHR on Cyp1a1 (ref 3)**	F: TATCCGGTATGGCTTCTTGC	R: CACCTTCAGGGTTAGGGTGA
**Mouse AHR on Il22**	F: ACAGTGATTTTCATGACTTCGCGTTCT	R: TCCCAGATAGCACCTGACAACTAGACT
**Mouse AHR on Ahr**	F: GTGTGTGCGCTCCCTTTGAC	R: GAGTCCGTCCACCAGTTCGTC

1. Vorrink, SU et al. (2013) Hypoxia perturbs aryl hydrocarbon receptor signaling and CYP1A1 expression induced by PCB 126 in human skin and liver-derived cell lines. *Toxicology and Applied Pharmacology* **274**, 408-416.
2. Gandhi R et al. (2010) Activation of the aryl hydrocarbon receptor induces human type 1 regulatory T cell–like and Foxp3+ regulatory T cells. *Nature Immunology* **11**, 846–853.
3. Amenya HZ et. al. (2016), Dioxin induces Ahr-dependent robust DNA demethylation of the Cyp1a1 promoter via Tdg in the mouse liver. *Scientific Reports* **6**, 34989.
